# Effect of Printing Process Parameters on the Shape Transformation Capability of 3D Printed Structures

**DOI:** 10.3390/polym14010117

**Published:** 2021-12-29

**Authors:** Matej Pivar, Diana Gregor-Svetec, Deja Muck

**Affiliations:** Chair of Information and Graphic Arts Technology, Faculty of Natural Sciences and Engineering, University of Ljubljana, Snežniška 5, 1000 Ljubljana, Slovenia; matej.pivar@ntf.uni-lj.si (M.P.); diana.gregor@ntf.uni-lj.si (D.G.-S.)

**Keywords:** additive manufacturing, 4D printing, PLA, TPU, printability, shape transformation

## Abstract

The aim of our research was to investigate and optimise the main 3D printing process parameters that directly or indirectly affect the shape transformation capability and to determine the optimal transformation conditions to achieve predicted extent, and accurate and reproducible transformations of 3D printed, shape-changing two-material structures based on PLA and TPU. The shape-changing structures were printed using the FDM technology. The influence of each printing parameter that affects the final printability of shape-changing structures is presented and studied. After optimising the 3D printing process parameters, the extent, accuracy and reproducibility of the shape transformation performance for four-layer structures were analysed. The shape transformation was performed in hot water at different activation temperatures. Through a careful selection of 3D printing process parameters and transformation conditions, the predicted extent, accuracy and good reproducibility of shape transformation for 3D printed structures were achieved. The accurate deposition of filaments in the layers was achieved by adjusting the printing speed, flow rate and cooling conditions of extruded filaments. The shape transformation capability of 3D printed structures with a defined shape and defined active segment dimensions was influenced by the relaxation of compressive and tensile residual stresses in deposited filaments in the printed layers of the active material and different activation temperatures of the transformation.

## 1. Introduction

Four-dimensional printing evolved from the 3D printing concept by incorporating the fourth dimension, i.e., the ability of the 3D printed object to change over time, to transform its geometry after being produced [1]. Today, 4D printing is becoming an increasingly interesting and widespread field of research. Some of the research is focused on the printing process parameters. Other research is more focused on the development of new, programmable printing materials that can change shape as a response to external stimuli. Based on the stimuli that can trigger a response, materials can be classified into thermo-responsive, magneto-responsive, chemo-responsive, photo-responsive or mechano-responsive materials [2].

One of the simplest options for 4D printing applications is the use of materials that are thermo-responsive, e.g., shape memory polymers (SMPs). SMPs have been used in 3D printing since 2013 [3]. These are dual-shape materials belonging to a group of actively moving polymers [4]. The shape memory effect is not an intrinsic property; it results from the combination of polymer morphology and specific processing. SMPs can be programmed into temporary shapes and return to their original shapes [5]. The mechanism of the shape memory effect (SME) can be described by two systems within the polymer, these being the net points and switching segments [6]. The net points, consisting of the more ordered, entangled or crystalline structure, act as the memory component of the polymer network that wants to return the SMP to its original shape [6]. The switching segments, consisting of less entangled structures, act to keep the SMP in its programmed shape [7]. Similarly to SMPs, the stimuli that induce time-dependent behaviour are temperature changes or water exposure [8].

The shape transformation depends on the residual stresses created during the 3D printing process in the thermoplastic materials. During the extrusion process, the thermoplastic materials are in a viscoelastic state—the high temperature enables the stretching and alignment of polymer chains in the direction of the material flow through the extrusion nozzle. After the material leaves the extrusion nozzle, it begins to cool and solidify. If the cooling is rapid, the polymer chains are forced to keep their extended state, which causes the development of internal stresses. When the 3D printed object is reheated above its glass transition temperature (Tg), the polymer chains start to rearrange, during which stress can be released, which causes shrinkage or changes in the shape of the 3D printed object [9,10,11,12].

Thermoplastic SMPs can be printed with the most widespread and cost-effective 3D printing technology, i.e., fused deposition modelling (FDM). The structure printed in the FDM process that will be capable of shape transformation is printed using one or a combination of two thermoplastic materials. In the first case, the shape transformation is controlled by a different orientation of deposited filament layers of the polymer [10,11,12,13,14], the so-called active material. In the second case, it is controlled by the multi-material structure, consisting of active and passive segments. For the active segment, a combination of polymers that differ in thermal transition temperatures and have different physical and mechanical properties is used [9,15]. One of the polymers, the active one, shrinks when heated above its Tg, and the second polymer, called the passive material, remains unchanged and serves only as a support for the active material to twist in a certain direction and plane. However, only one, i.e., the passive material, is used for the passive, inactive segment of the 3D printed structure. One article reported that single layer mono-material structures produce highly varying, unpredictable bending-twisting motions which are not desirable [15].

Polylactide (PLA) has shown shape memory properties based on the physical entanglements of polymer chains that maintain the structure of the 3D printed object [4,6,16,17]. After stretching into a temporary shape, the entanglements can recover to the unstretched state at Tg when the material becomes highly elastic [7]. Another thermoplastic material studied in 4D printing is thermoplastic polyurethane (TPU). The process parameters for filament extrusion and printing influencing shape memory behaviour were studied [1,18,19,20] using FDM to print a shape memory TPU for a thermoactivated self-folding part. Five parameters that are important for shape memory properties were studied, i.e., material/surface of the platform, material/surface temperature, printing speed, liquefier temperature and delay time for printing each layer. In our research, two different thermoplastics materials, i.e., PLA and TPU, were used to fabricate 3D printed shape-changing structures.

To be able to control the shape transformation, high printability and controlled transformation conditions must be achieved. The shape transformation capability is defined by extent, accuracy and reproducibility. The extent of transformation is defined by changing the angle or radius of the active segment of the 3D printed structure. It depends on the type and properties of the thermoplastic material; the shape of the active segment; 3D printing parameters that affect the generation of residual stresses; and transformation conditions defined by the type of the thermal stimulus, activation temperature and activation time. The shape transformation accuracy is defined by the transformation of the 3D printed structure in the predicted plane, depending on the direction of filament deposition. The reproducibility of the shape transformation is defined by the accuracy of the transformations of several active segments printed and transformed under the same conditions. The reproducibility and accuracy of the shape transformation depend on the properties of 3D printing filaments; printing process parameters which affect the accuracy of extruded filament deposition in layers; and transformation conditions. Among the research dealing with 3D printing filaments and printing process parameters, there is little about the accuracy and reproducibility of shape transformation, which is a topic of our research. We wanted to provide some insight into the optimisation of 3D printing process parameters that directly or indirectly affect the print quality, and consequently the quality of the shape transformation.

## 2. Materials and Methods

### 2.1. Materials

The thermoplastic materials used in the research were PLA and TPU, obtained from Plastika Trček (Ljubljana, Slovenia) in the form of monofilaments with a 1.75 mm diameter. These two commercial filaments were selected due to their different physical and mechanical properties, glass transition (Tg) and melting (Tm) temperature. White coloured PLA with Tg at around 60 °C and Tm between 150–160 °C was used for the fabrication of a part of an active segment, whereas black coloured TPU with the shore hardness A89, Tg bellow 0 °C and Tm at around 180 °C was used for the fabrication of the passive segment of 3D printed structures.

#### Drying of Thermoplastic Materials

Thermoplastic materials are mostly hygroscopic and must be dried before 3D printing to achieve good printability. Evaporation of moisture during extrusion causes the formation of pores in the printed object, which affects their geometry and mechanical performance [21,22,23]. To remove the initial moisture in filaments, drying in an oven was performed—PLA at 45 °C and TPU at 50 °C for 24 h. After the drying, the filaments were stored in an airtight container filled with a desiccant and fed to the 3D printer through a PTFE tube to protect them from environmental humidity.

### 2.2. 3D Printing

All 3D printed structures, hereinafter referred to as 3D test samples, were fabricated using a 3D printer ZMorph VX (ZMorph S.A., Wroclaw, Poland). Prior to the printing, 3D modelling of 3D test samples was performed with the software Blender. A G-code file to produce the 3D printed samples was generated from STL files using the software Slic3R.

Several printing process parameters were kept constant throughout the experiment to avoid their influence on shape transformation, among them the extrusion nozzle diameter and layer height. It was reported that with a higher layer height, lower residual stress and thus lower shrinking ratio are achieved [10,11,24]. In our study, an extrusion nozzle with the diameter of 300 µm was used and each layer was 200 µm in height.

To obtain high printability, it is necessary to calibrate the printer, to level the print bed and the extrusion nozzles height above it. If the printer is not calibrated, the nozzle cannot extrude the material properly, the first few layers can be compressed or may not stick to the platform [25]. To level the print bed, a ZMorph probe for a semi-automatic calibration was used in our case. To set the extrusion nozzle height, a single layer 3D test sample with the layer height of 200 µm was printed and its height was measured using a Holex digital caliper.

### 2.3. Optimisation of 3D Printing Process Parameters

The optimisation of printing process parameters is important to ensure the quality and dimensional accuracy of the printed object [26,27]. In our study, the flow rate, printing speed and cooling conditions were optimised to ensure high printability and reproducible residual stress formation in printed layers. The extrusion temperature was set to the lowest possible recommended by the producer. It was reported that with a lower extrusion temperature, a larger shrinkage in the filament length can be achieved [10]. The extrusion temperature for PLA and TPU was set to 195 and 230 °C, respectively.

#### 2.3.1. Printing Speed

The printing speed influences the shape transformation. Higher printing speed produces higher residual stress and as a result, higher shrinkage ratio [9,15,28,29,30]. Higher printing speed also leads to a less accurate extrusion process and lower print quality [31]. We assume that an increase in printing speed influences the accuracy of the shape transformation performance.

**Figure 1:**
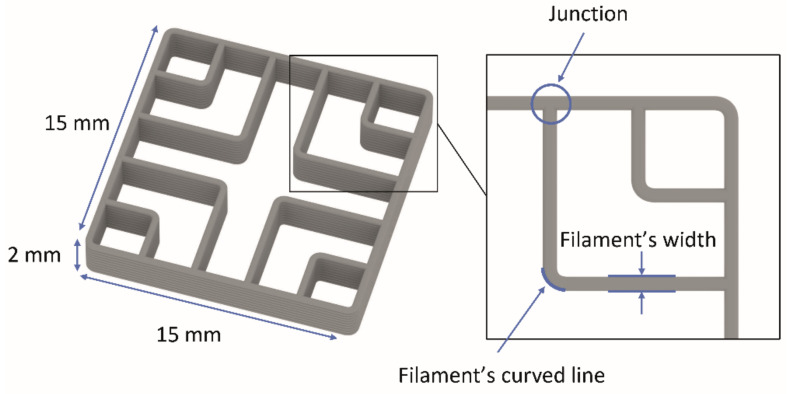
Scheme of 3D printed test sample.

To achieve the largest possible and most accurate shape transformation, the highest printing speed at which a satisfactory filament deposition accuracy was achieved was set as optimal speed. It was determined separately for both materials, based on the image analysis of 3D printed test samples (Figure 1). The recommended printing speeds for PLA and TPU are according to the producer 30–120 mm/s and 10–30 mm/s, respectively. To eliminate as many factors as possible which influence the shape transformation, the printing speed was set to the same value for both materials. Single wall 3D test samples, consisting of sequences of curved lines, were printed at 10, 20, 30 and 60 mm/s. The shape of curved lines, the width of filaments and precision of filament deposition at junctions were analysed with image analyses, as shown in Figure 1.

#### 2.3.2. Flow Rate

By varying the ratio of extrusion speed to printing speed, more or less material can be extruded, and if they are not appropriately synchronised, problems with the flow rate can occur [32]. In the case of over-extrusion, the defined shape of the printed object results in lower dimensional accuracy [33]. Deposited filaments are deformed and overlap, which affect the stress formation. Over-extrusion also increases the contact between the deposited filaments and the bonding between the polymer chains and provides high strength of the printed objects [34]. Another problem that occurs due to an insufficient material flow and orientation of deposited filaments are the voids between the deposited filaments, which reduce the strength of printed objects [34,35].

**Figure 2:**
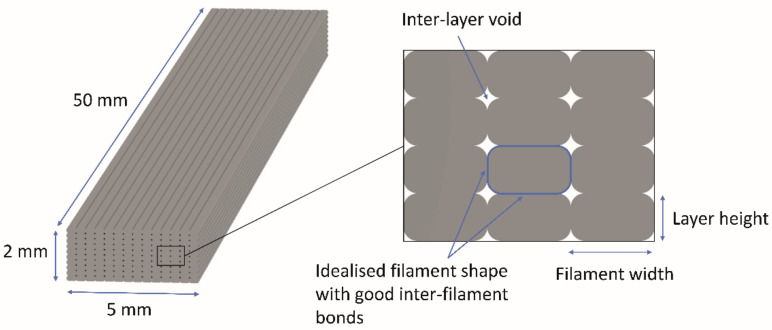
Scheme of theoretically ideal filament deposition.

For the shape-changing structures, the extruded filaments must be deposited as evenly and precisely as possible, with constant shape without overlapping and deformation, and the size of voids between them must be as small as possible. In Figure 2, a schematic representation of the theoretically ideal filament deposition is presented, which could be achieved by changing the extrusion width and flow rate at the same layer height. The extrusion width determines the position of each filament deposited, and the flow rate determines the amount of the extruded material. In our research, the extrusion width settings were left as default, only the flow rate was changed. The flow rate was controlled with an extrusion multiplier. The extrusion multiplier influences the amount of the material extruded in the unit of length travelled by the printhead with a given speed [33]. The extrusion multiplier value 1 means 100%, whereas 1.1 means 110% material flow. In our research, different values were studied to optimise filament deposition.

The flow rate was determined separately for PLA and TPU at a predetermined extrusion temperature and printing speed. The average value of the filament diameter measured with a digital caliper at several locations was entered into the slicing software to reduce the impact of the filament diameter deviation on flow rate. It was reported that the inconsistency across filament length will change the rate of material extrusion, resulting in dimensional imprecision [36]. For the determination of the flow rate, a 3D test sample of size 5 (x) × 50 (y) × 2 (z) mm was fabricated (Figure 2). A linear pattern with the infill density of 100%, oriented longitudinally in all layers was used.

The filament deposition was determined by determining the distribution and coverage of voids at the cross-sectional area of the 3D printed test specimens using the ImageJ software. The images, taken with a stereo microscope Nikon SMZ800 with a built-in high-resolution camera Nikon D850 (FX) (Nikon Europe, Amsterdam, Netherlands), with the lightning adapted to each material, were cropped to 2400 × 1400 pixels and converted to grayscale (8 bit). The images were processed using the Auto Local Threshold algorithm in ImageJ [37]. Based on a comparative analysis of different methods for deciding the threshold level (Bernsen, Contrast, Mean, Media, Midgrey, Niblack, Otsu, Phansalkar and Sauvola) and determining the radius of the pixel conversion area, the Midgrey method with the radius of 100 pixels for PLA and 70 pixels for TPU was chosen. With this method, the most accurate binary image of voids of all different sizes was achieved. The size distribution of voids was determined and is shown as a histogram.

#### 2.3.3. Cooling Conditions

The cooling conditions of the extruded filament are a very important printing process parameter, which influences the mechanical properties, visual quality and formation of residual stress. Immediately after the extruded filament leaves the extrusion nozzle, it begins to cool and solidify. The process of cooling is affected by the cooling fan speed parameter and the temperature of the extrusion nozzle, print bed and consequently ambient air. An article reported that the time interval between printing two adjacent layers also has a significant effect on the cooling and strength of the printed object [38]. Rapid cooling and fast solidification lead to limited chain diffusion and weak bonding between the deposited filaments and layers. In this case, lower strength is achieved [38]. In the case of insufficient cooling, the 3D printed object can deform [39]. During the printing, the surrounding air can heat up, which affects the solidification and formation of residual stress. It was reported that different environmental temperatures lead to different thermal gradients in the printed specimen. The specimen temperature decreases with the distance from the build plate and leads to different thermal expansions in different layers, causing warping defects. Cooler and higher layers shrink more due to a larger temperature difference between the specimen temperature and glass transition temperature of the material than lower and warmer layers [23].

In our research, the cooling fan speed was set to the average fan speed recommended by the producer, for PLA to 35% and for TPU to 20%, respectively. To control the cooling as much as possible and to eliminate the influence of the room air temperature, the printer enclosure was used.

In the works by Byoungkwon et al. [8] and Kačergis et al. [15], it was shown that the temperature of the print bed affects the shape transformation ability. Printing on a cooler print bed reduces the chain mobility, increases residual stress, leading to higher shape transformation performance. However, in the case of too low bed temperature, warping, bending of edges and even detachment of the first layer from the print bed can occur [25]. It was reported, that the optimal adhesion of printed objects to the print bed is achieved when the print bed is heated slightly above the Tg of the polymer material [40]. In our study, the temperature of the print bed was set to 60 °C (Tg of PLA) to achieve the dimensional accuracy of printed structures without warping and bending and good polymer chain diffusion between the deposited filaments. It was also reported that better adhesion is achieved by applying suitable glue [41]. To ensure adequate adhesion, the Dimafix spray adhesive (DIMA 3D, Valladolid, Spain) was used. To analyse the heating of the print bed, heat maps were taken with a Seek Thermal Reveal PRO camera (Seek Thermal Inc., Santa Barbara, CA, USA).

To achieve good accuracy and reproducibility of transformation, it is important that the cooling of the extruded filament stays constant throughout the printing process to create the reproducible residual stress in deposited filaments in all layers. To analyse the cooling of extruded filaments, a 3D test sample of size 200 (x) × 220 (y) × 0.8 (z) mm was prepared. A linear pattern with the infill density of 100%, oriented longitudinally in all layers, was used. The air temperature was measured in the vicinity of the 3D printed test sample at the distance of 1 to 2 mm. The temperature was measured with a FLUKE 287 instrument (Fluke Corporation, Everett, WA, USA), separately for PLA and TPU prints, and at printing both materials simultaneously. The measurements were performed within three hours with every minute reading.

### 2.4. Shape Transformation Capability

#### 2.4.1. Extent of Shape Transformation

**Figure 3:**
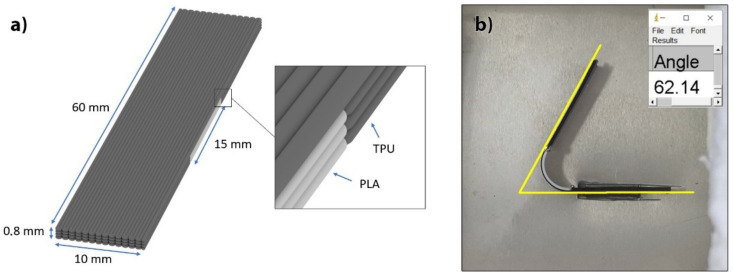
(**a**) Scheme of 3D printed test sample, (**b**) determination of extent (angle) of transformation.

The extent of the shape transformation was determined on the samples printed with optimised printing process parameters in water at different activation temperatures. It was reported that with a higher activation temperature, a larger shrinkage in the filament length can be achieved [10]. A four-layered 3D test sample with the size of 10 (x) × 60 (y) × 0.8 (z) mm and an active segment length of 15 mm (Figure 3a) was printed. In the study by Byoungkwon et al. [9] and by Shunsuke et al. [42], it was shown that the length of the active segment influences the shape transformation performance. With a larger active segment, a larger angle of transformation can be achieved. Our related preliminary research revealed that the optimal length of the active segment was 15 mm. The length of the passive segment on both sides of the active segment was set to 22.5 mm to be able to determine the transformation angle precisely (Figure 3b). The active segment was built from 3 layers of PLA and 1 layer of TPU, as also reported in the research by Byoungkwon et al. [9], since by increasing the number of layers, i.e., thickness of the active segment, the shape transformation performance deteriorates [28,43]. In our research, the passive segment of the 3D printed test sample consisted of 4 layers of TPU. A linear pattern with the infill density of 100%, oriented longitudinally in all layers, was used.

The shape transformation in water was performed in a bath with controlled heating, with ±1 °C accuracy. It was reported that hot water as a trigger provides uniform heating and high controllability with little gravitational effects, leading to better accuracy and repeatability; however, the triggering conditions must remain consistent [14]. The temperature during the transformation was monitored in the immediate vicinity of the 3D printed test sample. The activation temperatures used were 60, 70, 80 and 90 °C. The extent of the shape transformation, determined by measuring the transformation angle, was tested at different time intervals, i.e., 60 min at 60 °C, 15 min at 70 °C, 10 min at 80 °C and 5 min at 90 °C. The image analysis was applied to determine the change in the transformation angle. The images were captured with a Sony rx100V camera in a certain time interval/every 30 s (Figure 3b). From the measured angles, supplementary angles were determined and then applied to show the change in the angle as a function of the transformation time.

#### 2.4.2. Accuracy of Shape Transformation

In the study by Bona et al. [13], it was shown that the newly printed layer solidifies and shrinks on the previously deposited already hardened layer. Thus, the first printed layer undergoes compressive residual stress, and the upper layer undergoes tensile residual stress. When the stresses are released, the thermal deformation occurs in the opposite direction. The tensile deformation occurs in the first printed layers and the compressive deformation in the upper layers, resulting in sample bending. We assume that the differences in residual stresses affect the accuracy of the transformation and cause an unpredictable deformation during the transformation.

The influence of the optimisation of the 3D printing process and transformation conditions on the accuracy of the shape transformation capability was analysed. Our previous research has shown that a higher undesired deformation occurs at a thinner, longer and wider 3D printed structure. To eliminate as many factors as possible, 5-layered 3D test sample made entirely from PLA, with the size of 10 (x) × 50 (y) × 1 (z) mm was fabricated. A linear pattern with the infill density of 100%, oriented longitudinally in all layers, was used.

The accuracy of the shape transformation performance or the unpredictable deformation was determined on two sets of samples. The first set was composed from the test samples printed at optimised 3D printing process parameters, as previously discussed (hereafter called optimised printing or printing at optimised printing conditions). For comparison, the second set of samples was fabricated, using the same printing process parameter settings, though without 3D printer calibration (hereafter called non-optimised printing conditions). Afterwards, 3D printed test samples from both sets of samples were exposed to hot water at two different temperatures, i.e., 70 °C for 15 min and 90 °C for 5 min. For each series of testing, 5 specimens were prepared.

The dimensions of 3D printed test samples, before and after the exposure to hot water, were measured to 0.01 mm accuracy using a digital caliper to determine the directional strain (ε) and effective thermal expansion coefficient (α) in each printing direction (x, y and z) by using Equation (1).

(1) $$\alpha =\frac{\varepsilon }{\Delta T}=\frac{1}{\Delta T}\frac{\left(L_{f}-L_{0}\right)}{L_{0}}$$

where *L*_0_ and *L_f_* are the directional dimensions of printed samples before and after the exposure to hot water with the temperature change of Δ*T*.

In few cases, the dimensions could not be measured accurately with a caliper due to an excessive deformation of 3D printed structures. Therefore, test samples were captured with a Shining 3D OptimScan-5M inspection 3D scanner (SHINING 3D Technology, Hangzhou, China), with the scanning accuracy of 0.015 mm and their dimensions determined using the Blender software. The deflection in the vertical direction of printing (z direction) was determined using the 3D Scan-Optim software by fitting the 3D printed test specimen exposed to hot water to the reference sample (unexposed 3D printed test sample). 3D printed test samples were cut and the cross-sectional area was captured with a stereomicroscope Nikon SMZ800 with a built-in high-resolution camera Nikon D850 (FX). The surface coverage and circularity of voids and the dimensions of the deposited filaments in layers were determined with image analysis using the ImageJ software. The measurements were performed on ten randomly selected deposited filaments in each layer.

#### 2.4.3. Reproducibility of Shape Transformation

The reproducibility of the shape transformation was determined on the 3D printed test sample containing four identical active segments of 11.7 mm in length and passive segments of different lengths (10 and 20 mm). The previous testing of the length of the active segment and model prediction namely showed that with the length of 11.7 mm, the transformation angle of 90° could be reached. Five specimens were analysed. The transformation was performed in water at the temperature of 70 °C and a time of 15 min. After the shape transformation, the 3D printed test sample was captured with a high-resolution camera Nikon D850 (FX). The transformation angle was determined for each individual active segment with image analysis with the ImageJ software.

## 3. Results

### 3.1. Optimisation of Printing Process Parameters

#### 3.1.1. Printing Speed

**Figure 4:**
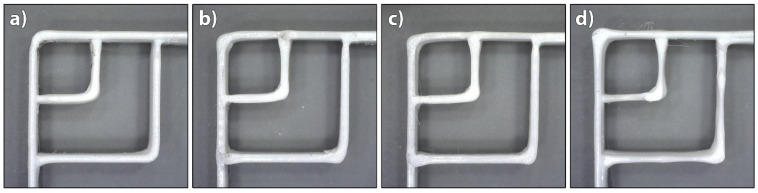
Images of 3D printed PLA test samples printed at different printing speeds: (**a**) 10 mm/s, (**b**) 20 mm/s, (**c**) 30 mm/s and (**d**) 60 mm/s.

**Figure 5:**
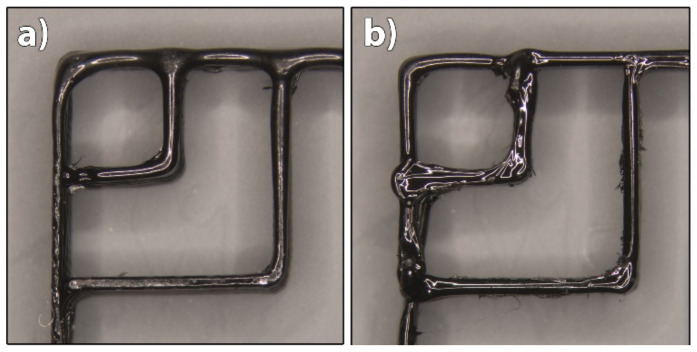
Images of 3D printed TPU test samples printed at different printing speeds: (**a**) 10 mm/s and (**b**) 20 mm/s.

Our research confirmed that the printing speed affects the deposition of filaments, and thus print quality. Irregularities occurred in the curved parts of the filaments, in the width of them and at junctions of two adjacent filaments due to the poor synchronisation of the printing speed and extrusion speed. The results of image analysis showed that satisfactory deposition of PLA filaments was achieved at printing speeds of 20–30 mm/s (Figure 4). At higher speeds, the extruded filaments deformed at the junctions and curved parts when deposited, and the width of the filaments in a layer was not the same and constant, as seen from Figure 4d. For TPU, however, irregularities occurred at the junction at the printing speed of just 20 mm/s (Figure 5b). The cause for this problem was oozing that could not be fixed due to their viscoelastic state at a certain extrusion temperature. Finally, the printing speed of 22 mm/s was determined as the maximum possible for TPU. At higher printing speeds, the feeding of the filament into the extrusion nozzle presented a problem due to the insufficient rigidity of the filament, interrupting the extrusion process. Elastomers, e.g., TPU, are prone to buckling during printing, which limits the printing speed. To have as few variables as possible that affect the deposition of extruded filaments when printing both materials at the same time, and to achieve the largest extent and most accurate transformation, both materials were printed at 22 mm/s.

#### 3.1.2. Flow Rate

**Figure 6:**
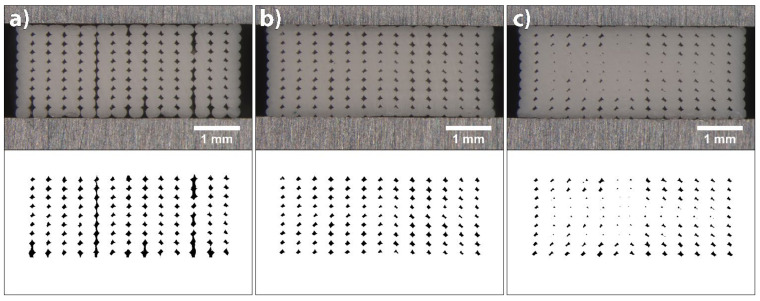
Images of cross-sectional area of 3D printed test samples from PLA and binary images belonging to them at extrusion multipliers: (**a**) 1, (**b**) 1.05 and (**c**) 1.1.

**Figure 7:**
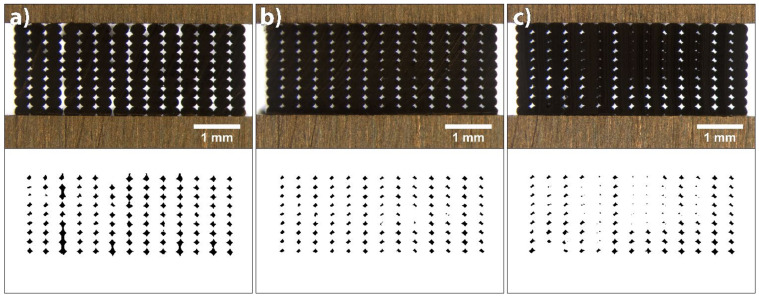
Images of cross-sectional area of 3D printed test samples from TPU and binary images belonging to them at extrusion multipliers: (**a**) 1.1, (**b**) 1.15 and (**c**) 1.2.

The influences of flow rate on the deposition of extruded filaments and size of voids in 3D printed structures were accessed at three different extrusion multiplier settings for both materials: 1.0 (default setting), 1.05 and 1.1 for PLA; and 1.1, 1.15 and 1.2 for TPU. The cross-sectional area of 3D printed test samples printed at different extrusion multipliers and their binary images are shown in Figure 6 for PLA and Figure 7 for TPU.

**Figure 8:**
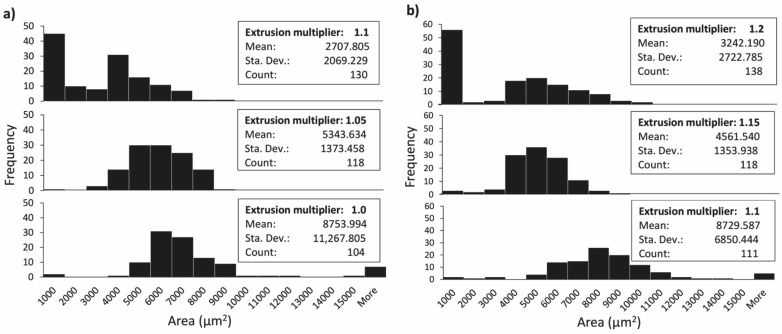
Size distribution of voids at different extrusion multipliers: (**a**) PLA and (**b**) TPU.

When printing PLA with default settings, the flow rate was too low. From the image of the cross-sectional area of the 3D printed test sample and its binary image, it can be seen that only limited contact and bonding between the filaments was present (Figure 6a). The total coverage of voids was determined to be 5.37%, and the average size was 8753.99 (±11,267.85) µm^2^. The size distribution histogram shown in Figure 8a is symmetrical, with a wide range and one outlier. This outlier was a void larger than 15,000 µm^2^, caused by an insufficient flow rate. Increasing the extrusion multiplier by 5% improved the bonding of extruded filaments; all filaments were joined and the voids were reduced in size (Figure 6b). The size distribution histogram remained symmetrical, without outliers. The total coverage of voids was determined to be 3.66%, and the average size was 5343.63 (±1373.46) µm^2^. In the case of an additional increase by 5%, however, the extruded filaments began to deform; the voids disappeared (Figure 6c). This phenomenon occurred in the central part of each 3D printed test sample due to the filament deposition within each layer, which was set from outside towards inside. The total coverage of voids was determined to be 2.03%, and the average size was 2707.81 (±2069.229) µm^2^. The size distribution histogram is asymmetric, and the outliers, below 1000 µm^2^ in size, stand out (Figure 8a). These outliers represent the voids in the central part; they started to close due to too much material being extruded.

The analysis of the size and distribution of voids showed that the optimal flow rate in the case of PLA was achieved with the extrusion multiplier of 1.05. The voids were mostly even in size and shape, which indicates even and precise deposition of the filaments in all layers. This is very important for the transformation accuracy and reproducibility of 3D printed structures. The deformation of the shape of individual filaments during the printing and their deposition when printing the active PLA segment influences the residual stress relaxation and leads to unwanted deformations of 3D printed structures.

When printing TPU at default settings and at the extrusion multiplier of 1.05, under-extrusion occurred, resulting in filaments being too far away to merge. Also, at the extrusion multiplier 1.1, the flow rate was too low, resulting in a limited contact and bonding between the filaments, as it is seen in Figure 7a. The total coverage of voids was determined to be 5.79% and the average size 8729.59 (±6850.44) µm^2^. The size distribution histogram shows a broad range and has a similar shape to the histogram for the PLA printed with default settings (Figure 8). In the case of a 5% increase in the flow rate, the total coverage of voids was reduced to 3.24% and the average size to 4561.54 (±1353.94) µm^2^. From the binary images and histogram, some smaller voids were clearly present, caused by the impurities in the voids and the deformation of the filaments (Figure 7b). By further increasing the flow rate, the size of voids reduced and the bonding of extruded filaments increased, whereas the filaments began to deform and deposited unevenly (Figure 7c). The total coverage of voids was determined to be 2.66%, and average size was 3242.19 (±2722.79) µm^2^. The size of voids was lower than 1000 µm^2^ in 40% of cases. As with PLA, the voids began to double due to the deformation of the filaments. The optimal flow rate in the case of TPU was achieved with the extrusion multiplier of 1.15. Though TPU is not an active material, the quantity of the extruded material is important, since it influences the transformation performance of the active material. A larger amount of the extruded material gives higher resistance to transformation and reduces the extent of transformation.

It is evident that the voids were quite large and could be reduced; however, in our case, this was not the priority. It was more important to obtain evenly deposited and interconnected filaments, with as little deformation as possible. A higher flow rate, besides reducing the size of voids and deforming the shape of the filaments, could lead to the dimensional inaccuracy of 3D printed structures. For the shape transformation ability, the ratio between the amounts of TPU and PLA materials is important. If the amount of TPU is higher, this could result in higher resistance and hinder the shape transformation performance. Therefore, the amount of the extruded material is even more important.

#### 3.1.3. Cooling Conditions

**Figure 9:**
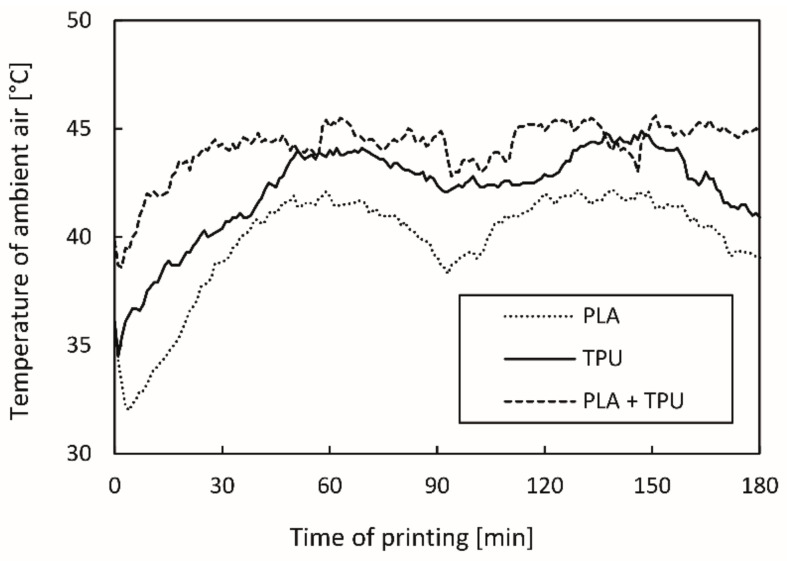
Temperature of ambient air in vicinity of 3D printed test sample as a function of time.

Measuring the ambient air temperature in the immediate vicinity of the 3D printed test specimen confirmed that the air temperature was below the Tg of the active PLA material, which prevented rapid residual stress relaxation during the printing process. Figure 9 shows the temperature of ambient air as a function of time for printing PLA and TPU separately, and PLA and TPU together.

**Figure 10:**
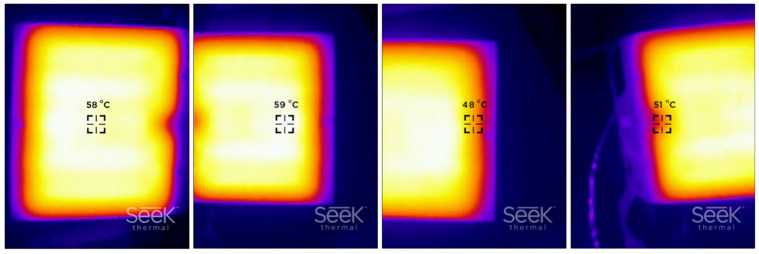
Heat maps of print bed.

As seen from Figure 9, the air temperature is slightly higher in the initial phase due to the heating of the extrusion nozzle before the printing when the cooling fan is switched off. As soon as the printing process begins, the fan is turned on and the temperature begins to drop to a certain point; then it starts to rise again before levelling out. A quite large fluctuation in the air temperature was observed, with a CV of 6.1% when printing PLA, slightly lower at that of TPU (5.1%), and below 3% during the simultaneous printing of both materials. The air temperature fluctuations depend on the position of the printhead and temperature of the working extrusion nozzle. The highest temperature was measured when the printhead was in the middle of the print bed. This is shown at around 60 and 135 min in the graph where individual PLA and TPU curves have the highest peaks. Along the sides of the print bed, the air temperature dropped significantly. This can be seen from the curves for the initial part of the printing at about 90 min, and for the final part of the printing, at about 180 min. The findings can also be confirmed from the series of images in Figure 10, which show the heat maps of the print bed, measured with a thermal camera. The heat maps show that the print bed was unevenly heated. The temperature was 59 °C in the warmest parts and 48 °C in the coldest parts (edges), although the print bed was heated to 60 °C. Such changes in temperature influence the formation of different residual stress in the active PLA material and consequently affect the shape transformation ability.

When printing a single material, a higher ambient air temperature (up to 44.9 °C) was measured for TPU, as the extrusion nozzle was heated to a higher temperature. Moreover, in this case, the air temperature did not exceed the Tg of the active PLA. When PLA and TPU were printed simultaneously, the air temperature was slightly higher, i.e., up to 45.6 °C.

### 3.2. Shape Transformation Capability

#### 3.2.1. Extent of Shape Transformation

**Figure 11:**
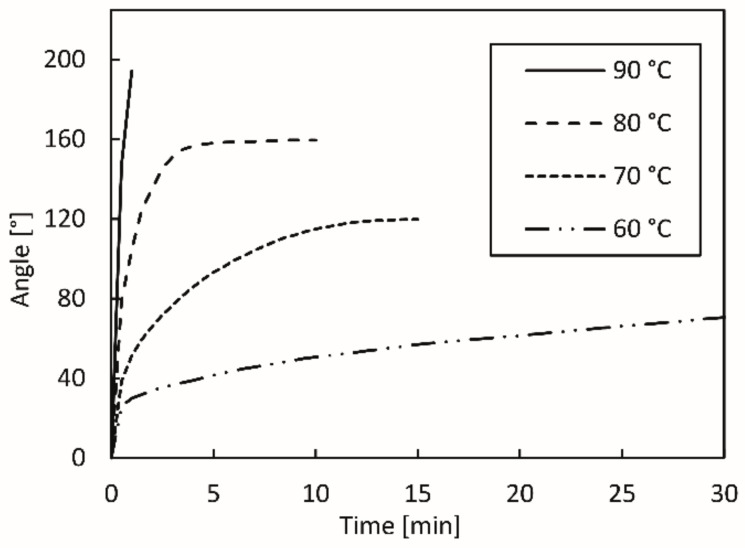
Transformation angle as a function of transformation time.

The research showed that the activation temperature influences the extent of shape transformation. In Figure 11, the changes in the transformation angle of the 3D printed test sample exposed to water as a function of the transformation time at different activation temperatures are presented. From the slopes and shapes of the curves, it can be deduced that the transformation rate and the achieved final angle were higher at higher activation temperatures. The shape transformation was quicker initially; then, depending on the activation temperature, it slowed down and stopped at a certain point, when the relaxation of residual stresses in all layers of the active part of the 3D printed test sample was reached.

**Figure 12:**
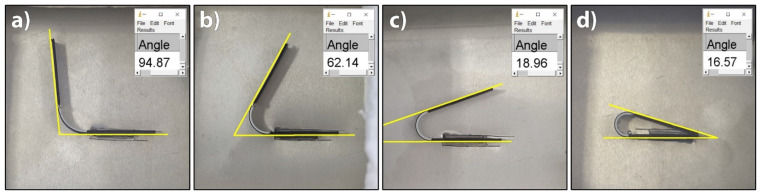
Determination of final transformation angles in water: (**a**) 60 °C, (**b**) 70 °C, (**c**) 80 °C and (**d**) 90 °C.

The lower the temperature of the water, the more time needed for the shape transformation. At 60 °C, the relaxation of residual stresses took about 60 min, although after 50 min, the change in the transformation angle was barely evident (about 1°). As the activation temperature rose, the relaxation of residual stresses accelerated and was completed earlier. At the activation temperature of 70 °C, it ended in 12 to 15 min; at 80 °C, after 8 min. When the temperature of 90 °C was used, the transformation was very fast, although it was difficult to determine the final transformational angle due to the length of the passive segment, which hindered the transformation (Figure 12d). From Table 1 and Figure 12, it is evident that a higher activation temperature causes a higher final transformation angle. For the water temperature of 60 °C, the transformation angle was around 83°, whereas it was around 195° for 90 °C water. The determination of the final transformation angles represent the basis for the determination of model prediction, which is the topic of another publication.

The results of the research showed that any fluctuations of the activation temperature have a strong effect on the relaxation of residual stresses and the final transformation angle. Therefore, it is very important that the activation temperature remains as constant as possible.

An example of the shape transformation of a 3D printed test sample exposed to water at 90 °C can be seen in the Video S1 (Supplementary Materials).

#### 3.2.2. Accuracy of Shape Transformation

**Figure 13:**
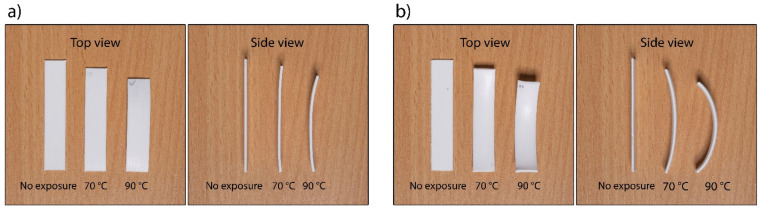
Test samples printed in (**a**) optimised printing conditions and (**b**) non-optimised printing conditions—no exposure (printed and not transformed), 70 and 90 °C (printed and transformed in water at 70 and 90 °C).

In Figure 13 and Table 2, the changes in the dimensions of the 3D printed test samples after the shape transformation in water are shown. It is evident that the thermal expansion coefficient (α) is negative in the longitudinal direction of 3D printing (y direction) and positive in the vertical direction (z direction) for all printing and shape transformation modes. This suggests that the residual stress relaxation is an anisotropic dimensional change of the 3D printed test samples. In the transverse direction (x direction), the thermal expansion coefficient is positive for optimised printing and negative for non-optimised printing. This means that different printing conditions affect the accuracy of the transformation. The coefficient of thermal expansion is higher at a higher activation temperature, meaning that the active PLA would shrink more, resulting in higher shape transformation. This was already found when determining the extent of the shape transformation of shape-changing structures.

**Figure 14:**
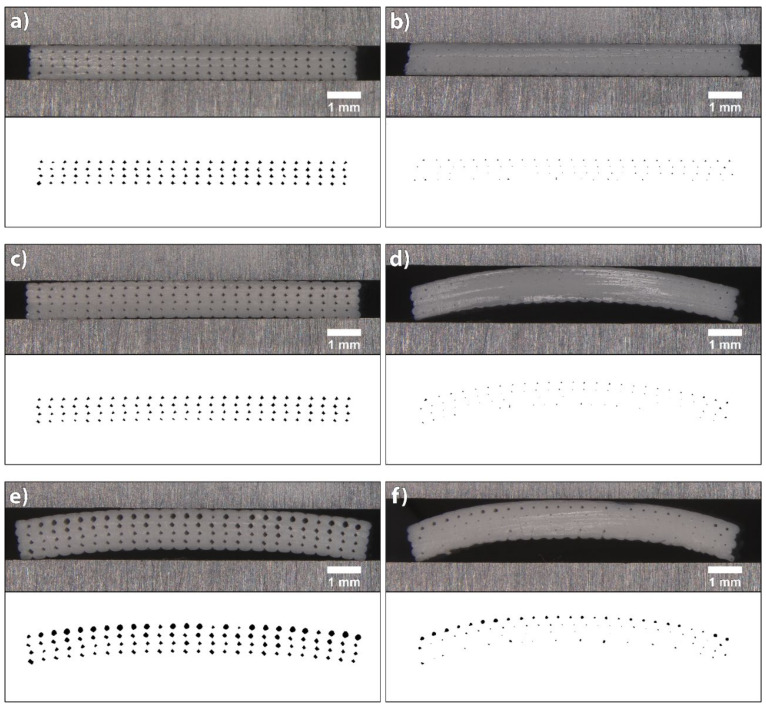
Cross-sectional areas and associated binary images of 3D printed test samples: (**a**) optimised printing conditions, (**b**) non-optimised printing conditions, (**c**) optimised printing and transformed at 70 °C, (**d**) non-optimised printing and transformed at 70 °C, (**e**) optimised printing and transformed at 90 °C, (**f**) non-optimised printing and transformed at 90 °C.

The binary images of the cross-sectional area photos are 2400 × 700 pixels in size and are shown in Figure 14. The total coverage of voids determined for the sample printed with optimised printing settings was 1.58%, and via non-optimised printing it was only 0.15%. 3D printing at optimised printing settings resulted in even and accurate deposition of filaments, with an even distribution of voids across the layers (Figure 14a). In non-optimised printing, the filaments were deposited more unevenly and inaccurately, resulting in smaller voids due to the deformation of the filaments. Some voids could not be captured even when adjusting contrast and brightness with global thresholding (Figure 14b).

After the shape transformation in water at the temperature of 70 °C (Figure 14c), the total coverage of voids in samples printed at optimised printing settings was 1.41%, which is only 0.17% less than the total coverage determined immediately after the printing. The difference is very small and is difficult to explain. The size of the voids may have been reduced due to the thermal expansion of the filaments in x and z directions, or the cause may have been the inhomogeneity of the sample or measurement uncertainty. Slight curvature or deformation of the 3D printed test sample in z direction was seen. The maximum measured deviation was 35.25 µm. With the deviation being so small, it had no impact on the transformation accuracy. The transformation in water at the temperature of 90 °C resulted in an increase of the total coverage of voids (3.38%). The voids were larger and of different shapes, and the filaments in the upper layers were deformed (Figure 14e). Furthermore, the curvature of the 3D printed test sample increased to 307.21 µm.

For non-optimised printing, the total levels of coverage of voids determined for samples before and after the transformation in water at the temperature of 70 °C were the same, i.e., 0.15%. Additionally, in this case, the smallest voids could not be captured after thresholding. However, a very large twist/bend occurred in the z direction of printing, with a maximum measured deviation of 624.57 µm (Figure 14d). The total coverage of voids of the sample exposed to water at the temperature of 90 °C increased to 0.58% due to the larger size of the voids. The curvature of the sample increased as well, the maximum measured deviation being 749.24 µm (Figure 14f).

**Figure 15:**
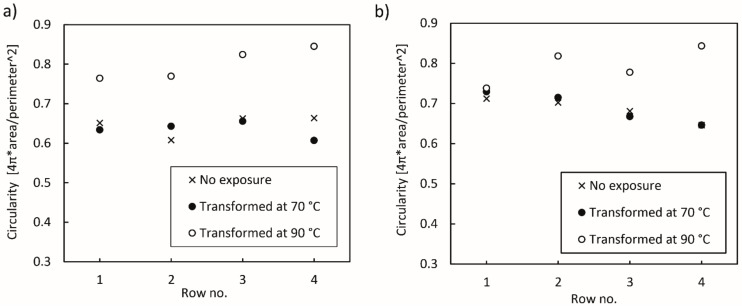
Circularity of voids in rows between layers determined on 3D printed test samples: (**a**) optimised printing conditions, (**b**) non-optimised printing conditions.

The analysis of the circularity of voids determined between the deposited layers in the samples printed and transformed under different conditions is shown in Figure 15. The shape of the voids after the transformation in water at 70 °C remained mostly the same as after the printing for both printing conditions, whereas at 90 °C, the voids took the form of a regular circle. For the samples transformed in water at 90 °C, the circularity of the voids increased after each deposited layer and peaked in the 4th row, i.e., between the 4th and 5th layer. Moreover, the size of voids increased from the 1st to the 4th row, as is clearly shown in Figure 14e.

**Figure 16:**
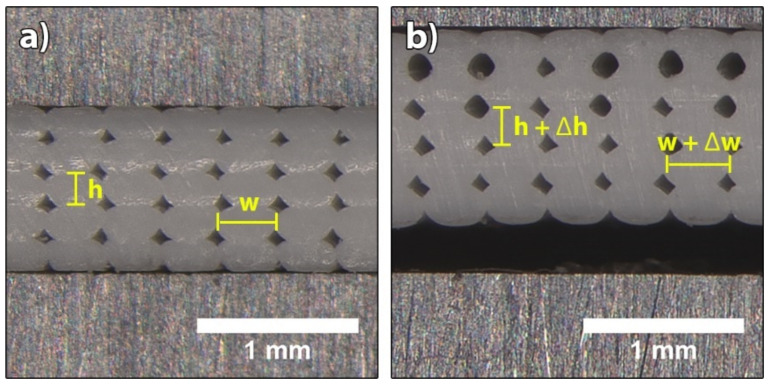
Images of the cross-section of a 3D printed sample for determination of height (h) and width (w) of deposited filaments: (**a**) no exposure, (**b**) after transformation at 90 °C.

**Figure 17:**
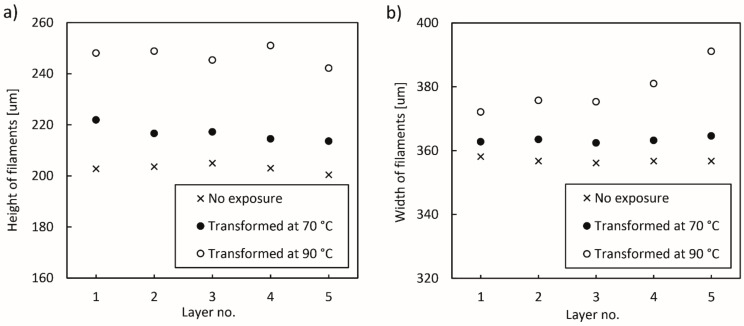
Dimensions of deposited filaments in layers determined in samples without exposure and after exposure to water at 70 and 90 °C: (**a**) heights of deposited filaments, (**b**) widths of deposited filaments.

The determination and results of the analysis of height (h) and width (w) of deposited filaments in the samples printed under optimised printing conditions and exposed to water are shown in Figure 16 and Figure 17. The analysis of the samples printed under non-optimised printing conditions could not be performed, as the filaments were too deformed for an accurate analysis. Nevertheless, we could visually determine that the filaments in the last deposited layer had the most regular shape, whereas the filaments in the first layer were the most deformed and compressed, most likely due to the incorrectly set offset of the extrusion nozzles from the print bed.

The average measured height of filaments determined in all five layers in the sample after the printing was 202.9 ± 5.24 µm, which indicates that the printing process parameters were optimised well, as the predicted height was 200 µm and the deviation was very small. After the transformation in water at 70 °C, the height of the filaments increased on average to 216.8 ± 6.45 µm, which coincides with a small thermal expansion coefficient (Table 2). After the transformation in water at 90 °C, the height increased to 247.1 ± 7.26 µm, which coincides with a higher thermal expansion coefficient. The cause for the thermal expansion is the compression of the extruded filament to the height of the layer. After leaving the extrusion nozzle with the diameter of 300 µm, the extruded filament must be compressed to the predicted layer height of 200 µm, whereby internal stresses are created inside the filament. During the shape transformation process, the residual stresses are released, the rearrangement of the polymer chains leads to changes in dimensions and the height of the filaments increases.

The widths of the filaments show similar values for the 3D printed test samples before and after the exposure to water at 70 °C. Differences, however, occurred in the 4th and 5th layer after the exposure to water at 90 °C. After the printing, the average width of the filaments of every layer was 356.9 ± 6.83 µm, which after the transformation at 70 °C increased slightly to 363.3 ± 6.84 µm, coinciding with the thermal expansion coefficient (Table 2). After the transformation at 90 °C, the width increased to 374.4 ± 8.54 µm in the first three layers, whereas in the 4th and 5th layers, the average widths were slightly higher, i.e., 381 ± 8.76 µm and 391.1 ± 6.66 µm, respectively. As a result, the upper two layers of the sample expanded more, causing the bending/twisting or deformation of the sample. The cause of the thermal expansion in the width of the filaments is related to the contraction of the filaments in the longitudinal direction. During the transformation/relaxation process, the filaments shrink in the longitudinal direction, and at the same time, the height and width of the filaments increase.

**Figure 18:**
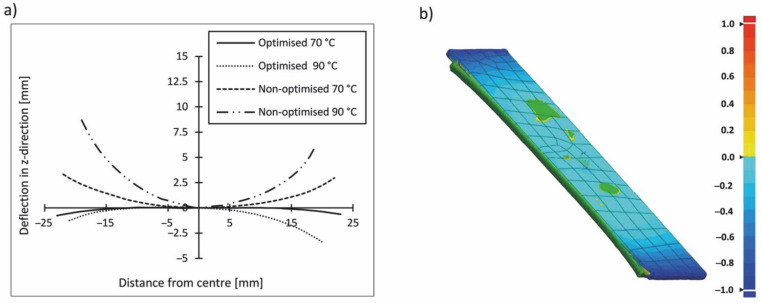
(**a**) Deflection in vertical direction of 3D printed test sample, (**b**) colour matching 3D plot of the sample transformed 70 °C with respect to reference.

The graph in Figure 18a shows the deflection of the 3D printed test samples in the vertical direction in regard to the xy-plane. The starting point of the coordinate system (z = 0, y = 0) represents the origin, and the upper surface of the undeformed specimen represents the reference. The curves on the graph, however, represent the deviations of the 3D printed test samples after the shape transformation in hot water from the xy-plane. The samples printed with the optimised 3D printing process parameters showed a negative deviation from the xy-plane, as they bent/twisted downward during the transformation. This can be explained by the difference in the temperature between filaments in adjacent layers. In the first layer, they were deposited on a relatively cold print bed (measured temperature 48–59 °C), so higher internal stresses were created in the material than in the remaining layers, where the temperature was higher and the cooling of the deposited filaments slower, enabling the residual stress relaxation. As a result, greater shrinkage of the first layer and twisting of the 3D printed test sample in the negative direction occurred.

The test samples printed with non-optimised printing, however, twisted in a positive direction. Bending in the positive direction is attributed to the inaccurate deposition of extruded filaments and their deformation. The deposition of the extruded filaments in non-optimised printing was not accurate enough; the filaments were differently stretched and deformed, which resulted in the differences in the temperature distribution, as is seen in the colour matching 3D plot (Figure 18b). The deflection from the xy-plane was higher when the printing process is not optimised and when it was performed at a higher activation temperature, as already determined with the extent of the shape transformation. At the temperature of 90 °C, the bending/twisting motion was not the same at both ends of specimen, suggesting a difference in the structure. The smallest deformation was detected in the optimised 3D printing process and at the activation temperature of 70 °C.

#### 3.2.3. Reproducibility of Shape Transformation

**Figure 19:**
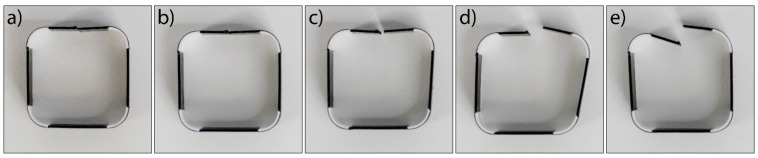
3D printed test samples after shape transformation in water at 70 °C: (**a**) and (**b**) ideal case; (**c**) small deflection of angle; (**d**) and (**e**) larger deflection of angle from 90°.

Figure 19 shows five 3D printed test samples transformed into the predicted shape of a square with rounded edges. The average transformation angle of the 20 active segments from specimens was 90.4° with the deviation of 5.9°. Two active segments deviated by more than 10°, and two between 6 and 8°. Different times for the transformation of the active segments in the same specimen were noticed, although the same final transformation angle was reached at the end of the shape transformation process. It is very important that the transformation reaches the final angle and stops there. If we could determine intermediate angles, due to different transformation speeds, we would also get different angles in the end. The reason for the transformations in some cases still ending with incorrect angles is still being investigated. We assume that the main causes were the unevenly heated heat bed, which affected the residual stresses, as explained in Section 3.1.3, and the time interval between the printing of two adjacent layers of a different active segment. The cause may also have been related to the geometry of the transformation of the 3D printed test sample and to water resistance during the transformation. Figure 19e shows that one active segment achieved a larger transformation angle than 90° if it was connected to a shorter passive element (10 mm), since it had less water resistance. Figure 19d shows the opposite. Additionally, some parts of the sample touched the bottom wall of the bath and inhibited the transformation.

## 4. Discussion

Printability is a very important factor influencing the shape transformation of 3D printed structures. Prior to the optimisation of the 3D printing process parameters, the calibration of the 3D printer was an important step to obtain an even and accurate distribution of filaments, with an even distribution of voids across the layers. By carefully selecting 3D printing process parameters, accuracy and good reproducibility of shape transformation for multi-material (PLA/TPU) 3D printed structures was obtained. High precision of filament deposition in layers was achieved by adjusting the printing speed, flow rate and cooling conditions of extruded filaments for both polymers printed simultaneously. The optimal printing speed which gave satisfactory deposition was 22 mm/s, which was the highest achievable for TPU and lowest feasible for PLA. For the optimal flow rate, the extrusion multiplier was selected with which the deposited filaments were as even in size and shape as possible, evenly deposited and spaced apart. The voids between them had to be as small as possible, and the connections between the deposited filaments as optimal as possible. The optimal flow rate was achieved with the extrusion multiplier of 1.05 for PLA and 1.15 for TPU. 3D printing was performed with a closed printer; the cooling speed was set to the average fan speed recommended by the producer; and the temperature of the print bed was set to 60 °C to ensure as constant cooling conditions as possible, to create the same residual stresses in the deposited filaments in all layers.

The shape transformation performance in hot water of a multi-material (PLA/TPU) 3D printed structure with a defined shape and defined active segment dimensions was substantially influenced by activation temperature. The extent and transformation rate increased with activation temperature and the uncontrolled deformation of the 3D printed structure. The activation temperature of 70 °C resulted in achieving the highest shape transformation accuracy.

To obtain the high transformation accuracy for multi-material (PLA/TPU) 3D printed shape-changing structures, the optimisation of printing process parameters is necessary, and exposure to water at the temperature of 70 °C is recommended. To achieve reproducibility in the case of printing PLA and TPU with a FDM 3D printer, we recommend using a print bed with evenly heating, or an unheated print bed and installing a local heating element to control and maintain constant ambient air temperature throughout the printing process to eliminate the formation of various residual stresses in the active PLA. The shape transformation must be performed in still water with the temperature remaining as constant as possible, and without the touching of other specimens or bath walls. In the case of touching the bottom of the bath, additives could be added to the water to increase the density of the water to reduce the effect of gravity.

Based on a pre-calibrated 3D printer and careful consideration of all the above parameters of printing flat four-layer structures with a combination of PLA and TPU materials, high-quality shape transformation was achieved. Based on repeatable empirical measurements under water exposure at 70 °C, the mathematical or analytical model of a bilayer beam composed of two materials with different thermal expansion coefficients can be established that predicts the transformation of printed structures that will change at different angles or radii of curvature by only changing the lengths of the active segments.

## Figures and Tables

**Table 1:** Transformation angles at different activation temperatures in hot water.

	Water Temperature			
	60 °C	70 °C	80 °C	90 °C
Angle min. [°]	80.6	117.9	157.4	194.0
Angle max. [°]	85.1	120.9	161.1	196.6
Average [°]	83.1	119.8	159.6	195.2
CV [%]	2.28	1.35	1.22	0.67

**Table 2:** Linear elongation and thermal expansion coefficient of 3D printed test samples in x, y and z directions.

Printing Conditions	Activation T [°C]	Direction	ε	α (×10^−3^ °C^−1^)
Optimised	70	Transverse (x)	0.013	0.285
		Longitudinal (y)	−0.064	−1.432
		Vertical (z)	0.064	1.417
	90	Transverse (x)	0.058	0.887
		Longitudinal (y)	−0.152	−2.342
		Vertical (z)	0.160	2.460
Non-optimised	70	Transverse (x)	−0.024	−0.539
		Longitudinal (y)	−0.072	−1.596
		Vertical (z)	0.084	1.858
	90	Transverse (x)	−0.021	−0.327
		Longitudinal (y)	−0.121	−1.869
		Vertical (z)	0.181	2.791

## Data Availability

Data are contained within the article.

## Supplementary Materials

The following supporting information can be downloaded at: https://www.mdpi.com/article/10.3390/polym14010117/s1, Video S1: An example of the shape transformation of a 3D printed test sample exposed to water at 90 °C.
